# Identification of a new Salmonella serovar – Salmonella Weitmar (8:z41:1,5)

**DOI:** 10.3205/id000102

**Published:** 2026-01-14

**Authors:** Carlos José Téllez-Castillo, Ann-Katrin Rekendt, Susanne Kollberg-Dix, Laura Pra-Mio, Claas Scharmann

**Affiliations:** 1 Praxis für Labormedizin und Mikrobiologie, Bochum, Germany

**Keywords:** Salmonella enterica, Salmonella Weitmar, foodborne gastroenteritis, new serovar, molecular diagnostics, MALDI-TOF MS, public health surveillance

## Abstract

We report the first human case of a novel *Salmonella enterica* serovar *Weitmar* (8:z41:1,5), isolated from a 41-year-old outpatient with acute diarrhea and fever in Bochum, Germany. Identification involved culture, multiplex PCR, MALDI-TOF MS, biochemical testing, and reference lab serotyping. The strain showed a unique antigenic profile and was confirmed by the WHO Collaborating Centre for Reference and Research on *Salmonella*. This case illustrates a routine but essential aspect of microbiological surveillance, highlighting how combined diagnostics and international collaboration support the reliable identification of novel *Salmonella serovars*.

Republication of: Téllez-Castillo CJ, Rekendt AK, Kollberg-Dix S, Pra-Mio L, Scharmann C. Identification of a new Salmonella serovar – Salmonella Scharmann (8:z41:1,5). GMS Infect Dis. 2025;13:Doc08. DOI: 10.3205/id000098

## Introduction

*Salmonella enterica* is a major cause of foodborne gastroenteritis worldwide, with significant public health implications [1]. The genus *Salmonella* comprises two species: *S. enterica* and *S. bongori*, and *S. enterica* is divided into six subspecies and more than 2,600 known serovars based on variations in O (somatic) and H (flagellar) antigens [2], [3]. Most human infectious diseases are caused by common serovars such as *S. enteritidis* and *S. typhimurium*, the identification of novel or rare serovars occasionally occurs in clinical settings [4], [5]. 

Only serovars belonging to *Salmonella enterica* subsp. *enterica* are officially assigned names, according to current nomenclature guidelines [2].

The detection and characterization of new *Salmonella* serovars are essential for public health surveillance, as they may indicate shifts in epidemiological trends, zoonotic transmission, or microbial adaptation [5], [6]. While serotyping remains the gold standard for *Salmonella* subtyping, molecular methods and proteomic tools like MALDI-TOF MS increasingly complement conventional diagnostics [6], [7]. 

In this report, we describe the first documented isolation and identification of a novel serovar, now officially named *Salmonella Weitmar* (8:z41:1,5), obtained from a human stool sample in an outpatient clinical setting. This finding highlights the critical role of advanced diagnostic tools and reference laboratories in recognizing and validating emerging pathogens. 

## Case description

### Clinical background

A 41-year-old patient presents to the outpatient clinic with acute watery diarrhoea and fever. A stool sample was sent along with requests testing of pathogenic bacteria, viruses, and parasites. The stool was noted to be of liquid consistency. The patient did not require hospital admission or antimicrobial treatment, as it was a self-limited case in the outpatient setting. 

### Diagnostic process

The stool sample was received in the laboratory and, due to its liquid consistency, was immediately processed for multiplex PCR. The BD MAX™ (Becton, Dickinson and Company, BD Life Sciences – Integrated Diagnostic Solutions, Sparks, MD, USA) Enteric Bacterial Panel included detection of bacterial pathogens (*Shigella*,* Salmonella*,* Yersinia*,* Campylobacter*, enteropathogenic* E. coli*), Enteric Viral Panel, detection of gastrointestinal virus (Rotavirus, Adenovirus, Astrovirus, Norovirus, Sapovirus), and Enteric Parasites Panel *(Giardia lamblia*,* Entamoeba histolytica*,* Cryptosporidium* spp.). In addition, Enzyme-Linked Immunosorbent Assay (ELISA) testing for *Clostridium difficile*, Glutamate Dehydrogenase (GDH) and Toxin A/B RIDASCREEN^®^ (R-Biopharm AG, Darmstadt, Germany), as well as antigen detection for *Helicobacter pylori* RIDASCREEN^®^ (R-Biopharm AG, Darmstadt, Germany), was performed. The PCR result was positive for *Salmonella*; all other targets tested negative. 

Following this, the sample was cultured on *Salmonella Shigella* Agar (SS Agar)/Xylose Lysine Deoxycholate (XLD) Agar Biplate Thermo Scientific™ (Thermo Fisher Scientific, Waltham, MA, USA), for detection of *Salmonella* and *Shigella* and inoculated into Selenite LBM^®^ Broth (BD Difco™, Becton, Dickinson and Company, Sparks, MD, USA), a selective enrichment medium). Incubation was carried out aerobically at 36±1°C for 24 and 48 hours on solid media, and for 24 hours in broth. 

After the growth of colonies with typical *Salmonella* morphology, flat, transparent to whitish colonies with a gray to black center appeared on SS agar and XLD agar. On XLD agar, small, transparent colonies within the red-colored medium, also with black centers, were observed. Species identification was performed using the MALDI-TOF Biotyper^®^ (Bruker Daltonics GmbH & Co. KG, Bremen, Germany), which confirmed the presence of *Salmonella* spp. with a score of 2.0. 

Subsequently, the colonies were inoculated into Kligler Iron Agar (Thermo Scientific™, Thermo Fisher Scientific, Waltham, MA, USA) for biochemical confirmation, followed by serological testing with specific antisera for *Salmonella* (Sifin Diagnostics GmbH, Berlin, Germany). 

The serological test results were as follows:

- Omni and Poly I: positive
- Group C: positive
- O7 antigen: negative
- O8 antigen: positive
- All H antigens: negative

After 24 hours of incubation at 36±1°C, the Kligler Iron Agar reaction was consistent with *Salmonella* spp.:

- Slant/butt reaction: red/yellow
- H2S production: positive
- Gas production: negative

Due to the absence of detectable H antigens, the isolate was sent to a partner laboratory (Institute for Hygiene and Environment (HU) Hamburg, Germany) for advanced typing. 

There, the strain was identified as *Salmonella* 8,20:z41:1,5, a previously unclassified serovar within *Salmonella enterica* subspecies *enterica*. The isolate was subsequently forwarded to the WHO Collaborating Centre for Reference and Research on *Salmonella* (Paris, France) for official confirmation. 

Following review, the strain was validated and formally recognized as a new serovar, named: *Salmonella Weitmar* (8:z41:1,5).

## Discussion

This case demonstrates the evolving landscape of enteric pathogen diagnostics and the critical role of advanced laboratory techniques in the identification of novel microbial strains. The rapid detection of *Salmonella* spp. via multiplex PCR allowed for swift initiation of culture-based methods, which remain essential for confirming pathogen viability, enabling antimicrobial susceptibility testing, and facilitating downstream phenotypic and genotypic analyses [8], [9]. 

While conventional serotyping remains the cornerstone of *Salmonella* classification, its limitations become evident in atypical or novel antigenic profiles [10], [11]. The isolate in this case failed to express detectable H antigens, rendering it untypeable by routine slide agglutination methods. This underscores the diagnostic gap faced in frontline laboratories when encountering antigenically deviant strains [2], [3], [9]. Referral to a reference centre enabled the application of extended typing methods, ultimately identifying the isolate as *Salmonella enterica* subsp. *enterica* serovar 8,20:z41:1,5 a previously unrecognized antigenic formula. Following rigorous characterization and confirmation, the isolate was designated *Salmonella Weitmar* by the WHO Collaborating Centre for Reference and Research on *Salmonella* [2]. The emergence of *Salmonella Weitmar* raises important questions regarding its epidemiological and clinical significance. Is this serovar regionally endemic, or is its identification merely a reflection of improved diagnostic resolution? The current case was selflimiting and did not require antimicrobial therapy, yet its pathogenic potential, environmental reservoir, transmission route, and resistance profile remain unknown. These questions warrant further investigation through genomic surveillance, animal and food source tracking, and integration into national and international monitoring systems [10], [11]. 

The case also highlights the pivotal role of collaborative networks and reference laboratories in global pathogen surveillance [11], [12]. Without the capacity to refer ambiguous isolates for expert analysis, novel serovars may go unrecognized, hindering the ability to detect emerging trends, assess zoonotic risks, or respond to outbreaks in real time [1], [12]. 

Finally, this report emphasizes the synergy between modern molecular technologies, such as MALDI-TOF MS and multiplex PCR, and classical microbiological techniques [6], [7], [8], [9]. Together, they provide a comprehensive diagnostic approach that supports both routine clinical decision-making and public health surveillance. 

In conclusion, the identification of *Salmonella Weitmar* reflects the value of integrating advanced diagnostics, careful clinical observation, and international cooperation. While the detection of new serovars is a routine part of reference laboratory work, the recurrent identification of previously uncharacterized *Salmonella* variants reflects the ongoing genetic diversification within the species. Such findings emphasize the importance of continuous surveillance and characterization to enhance understanding of *Salmonella* population dynamics and potential implications for public and animal health.

## Conclusions

This case illustrates the relevance of integrating molecular and classical microbiological methods, combined with international reference collaboration, for reliable identification and validation of novel *Salmonella* serovars. Maintaining diagnostic vigilance and supporting global surveillance systems is important to ensure their recognition and documentation within routine public health monitoring.

## Notes

### Author’s ORCID

Carlos José Téllez-Castillo: 0000-0003-3722-2830

### Author contributions

Conceptualization (CJTC); methodology (CJTC and AKR); writing and original draft preparation (CJTC and AKR); review and editing (CJTC, CS, and all authors); supervision (CJTC and CS). All authors have read and approved the final version of the manuscript. 

### Ethics statement

This case report does not contain any personal data or identifiable patient information. The isolate characterization was performed on anonymized clinical material as part of routine diagnostic procedures, and no specific informed consent was required. 

### Acknowledgements

We thank the Institute for Hygiene and Environment Hamburg (Dr. med. Judith Overhoff) and the WHO Collaborating Centre for Reference and Research on *Salmonella* for their support in strain characterization. We also wish to acknowledge the treating physician, Dr. med. Regina Mertens, for her clinical management of the patient. 

### Competing interests

The authors declare that they have no competing interests.
